# Integrated FT-IR and SPME-GC-MS Evaluation of Toxic Fire Effluents from Plastics Containing Brominated Flame Retardants

**DOI:** 10.3390/ma19132734

**Published:** 2026-06-26

**Authors:** Monika Borucka, Kamila Mizera, Jan Przybysz, Agnieszka Gajek

**Affiliations:** Central Institute for Labour Protection—National Research Institute (CIOP-PIB), Czerniakowska St. 16, 00-701 Warsaw, Poland; kamiz@ciop.pl (K.M.); japrz@ciop.pl (J.P.); aggaj@ciop.pl (A.G.)

**Keywords:** fire effluents analysis, steady-state tube furnace, fire toxicity, novel brominated flame retardants, FT-IR and SPME-GC-MS analysis, FED

## Abstract

**Highlights:**

- Replacement of brominated flame retardants significantly alters the toxicity of fire effluents.
- High thermal stability does not prevent the formation of highly toxic combustion products.
- Integrated FT-IR and SPME-GC-MS analysis was applied under realistic fire conditions.
- Fire-retarded epoxy systems show a significant increase in FED-based toxic hazard.
- Formation of brominated aromatics drives raised combustion toxicity.

**Abstract:**

Despite their high effectiveness in reducing material flammability, modern brominated flame retardants (BFRs) remain poorly understood with respect to the toxic substances they generate during combustion. BFRs such as 1,2-bis(pentabromodiphenyl)ethane (DBDPE) and tetrabromophthalate diol (PHT4-DIOL) have been introduced following the limitations on legacy brominated additives. However, their thermal decomposition pathways and toxic product emission profiles under real fire conditions remain poorly characterized. Exposure to elevated temperatures may promote the formation of halogenated toxicants and environmentally persistent compounds, raising concerns that extend beyond conventional fire-safety performance. The combustion behavior of DBDPE-, PHT4-DIOL-, and BFR-containing epoxy resins was investigated using a steady-state tube furnace designed to reproduce realistic fire scenarios. Controlled temperature and ventilation conditions were applied to simulate representative stages of fire. Combustion emissions were comprehensively characterized using Fourier transform infrared spectroscopy (FT-IR) to analyze asphyxiant and irritant gases and solid-phase microextraction gas chromatography–mass spectrometry (SPME-GC-MS) for volatile and semi-volatile organic compounds. The results presented that the incorporation of BFRs substantially altered combustion emission profiles, promoting the formation of brominated and mixed-halogenated species alongside toxic gaseous products. Significant differences in the composition and distribution of combustion byproducts were observed between non-modified and BFR-containing materials, indicating that the environmental and toxicological consequences of these additives cannot be adequately assessed solely through flammability-reduction metrics. These conclusions provide new knowledge of the environmental impacts of brominated flame retardants and highlight the importance of integrated fire-safety assessment strategies that simultaneously consider flame-inhibition efficiency, combustion toxicity, and environmental persistence.

## 1. Introduction

Epoxy resin (EP) is a crucial engineered polymer that has garnered significant attention in recent years due to its versatility, industrial applications, and economic benefits. Known for its remarkable characteristics, including strong adhesion to various substrates, resistance to moisture, solvents, and chemicals, and outstanding mechanical and electronic properties, epoxy resin is widely used across diverse industries. Its applications range from adhesives, coatings, laminates, and encapsulation to electronic insulation, construction materials, industrial flooring, marine systems, foams, composites, and aerospace components [1].

Despite their extensive use and benefits, the flammability of commercially available epoxy resins poses challenges in high-temperature environments. To overcome this limitation, epoxy resins are modified with effective flame-retardant additives containing bromine, phosphorus, silicon, or nitrogen to enhance thermal stability and broaden their practical applications. Additionally, developments in resin formulations, such as bio-based variants and low-VOC options, are stimulating innovation to meet eco-friendly targets and regulatory requirements. These modifications have made epoxy resins necessary in high-efficiency applications such as lightweight automotive components, durable electronics, and aerospace structures [1,2,3,4,5].

Several studies have demonstrated that brominated phenols [6], polybrominated dibenzo-p-dioxins (PBDD) and dibenzofurans (PBDF) [6,7,8], and other brominated organic products [8,9] may be formed during the combustion of brominated flame retardants. Bromine-containing flame retardants may produce corrosive, obscuring smoke and release toxic halogenated compounds during combustion, entailing significant risks toward environmental sustainability and human health [7]. Moreover, compounds such as hexabromocyclododecanes (HBCDs), polybrominated diphenyl ethers (PBDEs), and polybrominated biphenyls (PBBs) have been progressively banned or phased out due to their persistence in the environment, their ability to bioaccumulate, and their harmful impacts on both ecosystems and human health [10,11,12].

To reduce these drawbacks, novel flame-retardant materials are being developed [12]. One of the important new brominated flame retardants is 1,2-bis(pentabromodiphenyl)ethane (DBDPE) [10,12,13,14,15,16,17,18,19,20].

**Figure 1:**
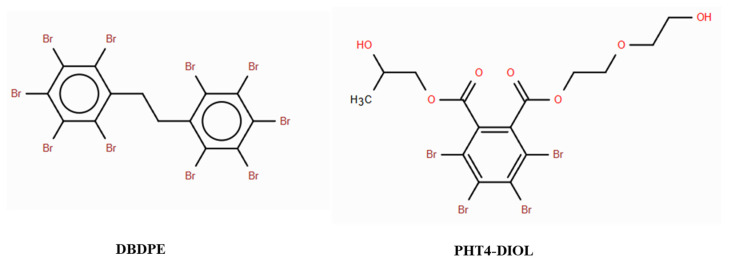
Chemical structures of: 1,2-bis(pentabromodiphenyl)ethane (DBDPE) and tetrabromophthalate diol (PHT4-DIOL).

The structure of 1,2-bis(pentabromodiphenyl)ethane and tetrabromophthalate diol is presented in Figure 1. Because of its stability, DBDPE was introduced as an alternative to commercial DBDE in the early 1990s, and it currently accounts for 83% of the BFRs in e-waste and plastics [18,19]. Concurrently, numerous studies have examined the thermal degradation of plastics containing DBDPE [14,15,17,18,19,20]. Previous studies have primarily focused on the pyrolysis mechanisms of brominated flame retardants; however, few studies have investigated the actual emissions of toxic fire effluents generated during the combustion of polymer materials containing these additives under conditions that simulate real fire scenarios. For example, Liu et al. [18] investigated the thermochemical degradation of electronic-waste plastics containing DBDPE using an online thermogravimetric–Fourier transform infrared–mass spectrometry (TG-FTIR-MS) system. The results showed that the debromination of DBDPE produced low-brominated diphenyl ethanes, which were subsequently further decomposed into brominated monoaromatic compounds and bromine radicals. The studies conducted by Yang et al. [19], based on previous research [18], discuss the potential formation of polybrominated dibenzo-p-dioxins and dibenzofurans (PBDD/Fs) during the pyrolysis of DBDPE. Moreover, Grause et al. [20] found that the main products of thermal degradation of high-impact polystyrene containing DBDPE were brominated toluenes, diphenylethanes, and brominated phenanthrenes using thermogravimetry coupled with mass spectroscopy (TG-MS). Long et al. [14] studied the thermal degradation of DBDPE and its pyrolysis mechanism in the presence of H radicals using quantum-chemical methods. The degradation of DBDPE with H radicals was primarily characterized by debromination, with the main pyrolysis products being low-brominated diphenyl ethanes, brominated monoaromatic compounds, brominated phenanthrenes, and HBr. PHT4-diol is widely used in the manufacturing of rigid polyurethane foam (RPUF) due to its high bromine content (68.9%) and compatibility with polyurethane chemistry [21,22]. Despite its industrial prevalence, critical knowledge gaps persist regarding its thermal stability and the byproducts formed during thermal decomposition or combustion. According to the literature, TGA data for PHT4-Diol indicate that the compound exhibits thermal degradation at moderate temperatures, whereas rapid and extensive decomposition occurs above approximately 300 °C [23]. Although direct studies on the pyrolysis mechanism of PHT4-diol are scarce, the available literature on structurally related brominated flame retardants, such as tetrabromobisphenol A, indicates that the main thermal decomposition products include HBr, brominated phenols, brominated aromatic compounds, brominated benzene derivatives, as well as soot and char [6].

In the literature, there is a lack of scientific studies on the products formed during the thermal decomposition and combustion of selected brominated flame retardants under real fire conditions.

Simultaneously, fires involving these materials are common due to their widespread use. One example of an incident involving flame-retardant polymeric plastics is the 2017 Grenfell Tower fire in London. As a result of this building fire, 72 people lost their lives. On 4 September 2024, the final investigation report on the fire was published, which identified the medical cause of death for most victims as inhalation of fire smoke, particularly asphyxiating gases such as CO and HCN [24]. It was shown that fire deaths are strongly associated with smoke toxicity in living rooms and bedrooms, so fires involving flame-retarded upholstered furniture may be one of the probable contributing causes of death [25,26]. This example confirms how important it is to analyze the thermal decomposition and combustion processes of materials under conditions as close as possible to real fire scenarios, since the products of their thermal degradation and combustion largely determine the toxicity of smoke and the resulting threat to human life. It is also worth mentioning that this fire caused significant environmental contamination. Soil samples collected from the surrounding area revealed high concentrations of benzene, benzo(a)pyrene, phosphorus, and polycyclic aromatic hydrocarbons [27,28]. Airborne brominated dioxins and furans have been detected in real fires, confirming that these pollutants pose practical concerns for both fireground environments and the surrounding atmosphere. Firefighters are routinely exposed to these compounds, and such toxicants have been linked to increased cancer rates and long-term health impacts [29,30,31].

Studying the toxic products present in fire effluents emitted under combustion conditions that reflect real fire scenarios is valuable, as it provides important knowledge about the hazards posed by specific materials to human health and life, as well as to the natural environment. Hence, the purpose of this work is to assess the impact of flame-retardant additives on the composition of gas and smoke mixtures that may form during the combustion of these materials, which can occur, for example, during accidental fires. Ultimately, addressing this gap is crucial to fully understanding the environmental burden of these materials, to ensure the development of safer fire-safety regulations, and to drive innovation toward lower-toxicity polymer formulations.

In this work, we investigated the asphyxiants, irritants, and volatile and semi-volatile organic compounds released in fire effluents during the thermal degradation of brominated polymer flame retardants. The study encompassed both the flame retardants themselves—1,2-bis(pentabromodiphenyl)ethane and tetrabromophthalate diol—and epoxy resins incorporating these additives to reflect realistic material use. A steady-state tube furnace was employed to simulate real fire scenarios under controlled conditions, thereby generating toxic combustion products. Asphyxiant and irritant gases were quantified using Fourier transform infrared (FT-IR) spectroscopy, while solid-phase microextraction (SPME) followed by gas chromatography–mass spectrometry (GC-MS) was applied to identify volatile and semi-volatile organic emissions. Altogether, this integrated analytical approach provides a comprehensive characterization of emission profiles from brominated flame-retarded systems, contributing to a better understanding of their environmental and health relevance under fire conditions.

## 2. Materials and Methods

### 2.1. Materials

For testing purposes, samples of the studied materials were prepared: epoxy resin (EP), epoxy resin containing an additive of 1,2-bis(pentabromo-phenyl)ethane (EP + DBDPE), and epoxy resin containing an additive of tetrabromophthalate diol (EP + PHT4-DIOL).

The substrates used for the polymer matrix were Epidian 6 (epoxy number 0.51–0.54 mol/100g, viscosity 10,000–15,000 mPas) and curing agent IDA (amine number 250–350 mg KOH/g, viscosity 150–300 mPas) purchased from P.H.U. Krisko (Lublin, Poland). The weight ratio of epoxy resin to curing agent was 1:0.5. The flame retardants were used at a concentration of 20% by weight of the total mixture. Based on our previous research [32,33], we determined this level to be optimal for achieving flame retardancy while maintaining the material’s performance properties.

Epoxy resin Epidian 6 and the flame retardant were mixed using a high-speed mechanical stirrer, proLAB 075 (GlobimiX Ltd., Zabkowice Slaskie, Poland), with a water jacket and a vacuum venting system. The stirring was carried out at rotational speeds of 2000, 3000, and 5000 rpm for approximately 1 min each. Next, the curing agent IDA was introduced, the mixture was stirred again at 2000 rpm for approximately 3 min, and then poured into the molds. The samples were cured at room temperature and post-cured at 70 °C for 3 h. Samples of unmodified epoxy resin (EP) were also prepared as described above.

### 2.2. Methods

The steady-state tube furnace (Purser furnace [34]) has been specifically designed to simulate real fire scenarios under controlled oxygen-deficient conditions, thereby enabling the generation of toxic combustion products. The method was used to model fire stages in accordance with the standard ISO 19706 [35].

The test series was started under well-ventilated conditions (Fire Stage 2). For the flame-retardant samples, no flaming combustion was observed at 650 °C. Therefore, in accordance with the recommendations of the ISO 19700 [34,36], the decomposition temperature was gradually increased. However, even at 825 °C, the samples still did not undergo flaming combustion. In contrast, the epoxy resin samples exhibited flaming combustion at 650 °C, with an initial airflow rate of 10 L/min. Additionally, the oxygen depletion (D_O2_), calculated from the average oxygen concentration in the mixing chamber, met the standard’s assumptions. Therefore, under these conditions, the epoxy resin samples underwent combustion that was representative of Fire Stage 2 conditions. Detailed information regarding the experimental conditions used during the measurements is summarized in Table 1. All measurements were repeated three times to ensure reproducibility.

The concentrations of carbon monoxide (CO), hydrogen cyanide (HCN), formaldehyde (CH_2_O), nitrogen oxides (NO_x_), sulphur dioxide (SO_2_), ammonia (NH_3_) and light hydrocarbons (methane (CH_4_), ethane (C_2_H_6_), propane (C_3_H_8_), ethylene (C_2_H_4_), hexane (C_6_H_14_)) in fire effluents were analyzed through Fourier transform infrared spectroscopy (FT-IR) with the Gasmet DX4000 portable analyzer (Gasmet Technologies Oy, Vantaa, Finland). Sampling involved extracting combustion gases from the furnace’s mixing chamber via a PSP4000-H probe (M&C TechGroup, Ratingen, Germany) interfaced with Gasmet’s portable collection system. The analysis was performed continuously throughout each measurement.

To assess the effects of fire effluents released during the combustion of the tested materials, the Fractional Effective Dose (FED) was calculated. The general FED equation is:

(1) $$FED=\int_{t1}^{t2}\sum_{i=1}^{n}\frac{Ci}{{\left(Ct\right)}_{i}}∆t$$

where *Ci* is the average concentration of a dose-related toxicant such as an asphyxiant gas ‘*i*’ over the chosen time increment; *∆t* is the chosen time increment, expressed in minutes (min); and (*Ct*)*_i_* is the specific exposure dose expressed as concentration minutes [34,35,37]. In the study, the estimate was based on the additive effects of CO, NO_2_, NO, SO_2_, HCN, and CHOH, accounting for their known toxicological interactions in fire scenarios. The calculations did not include emissions of NH_3_ and HCl, as these gases were not detected in the fire effluents.

Volatile and semi-volatile organic compounds released during the thermal decomposition of selected materials in fire effluents were sampled using a carboxen/polydimethylsiloxane (CAR/PDMS) solid-phase microextraction (SPME) fiber (Supelco, Bellefonte, PA, USA) [38]. Before sampling, the fiber was conditioned in the gas chromatograph (GC) injection port according to the manufacturer’s instructions. The SPME device was exposed to the effluent from the mixing chamber for 5 min and immediately thermal-desorbed in the GC injector port (at 270 °C) for analysis. The samples were collected during the steady-state period of decomposition for each material, as specified in Table 1. The volatile and semi-volatile products were analyzed using a gas chromatograph (GC 7890A, Agilent Technologies, Hanover, MD, USA) coupled to a mass spectrometer (MSD 5975, Agilent Technologies, Hanover, MD). Chromatographic separation was achieved on a HP-5MS fused-silica capillary column (30 m × 0.25 mm × 0.25 μm film thickness) using helium as the carrier gas at 1 mL/min. The oven temperature was maintained at 40 °C for 3 min, increased by 5 °C/min to 75 °C and held for 10 min, then increased by 10 °C/min to 280 °C and held for 5 min. The MSD was operated in electron-impact (70 eV) mode in scan mode (25–450 *m*/*z*). Chromatographic peaks were identified by matching mass spectra against the NIST MS Library, with a confidence threshold > 90%. To compare the variation of the identified products, the components were quantified by normalizing their peak integration areas. The results are presented in a quantitative format to provide an approximate indication of the concentrations of these substances. The total identified peak areas were normalized to 100%, and the relative abundance of each compound was defined as its proportion of the total peak area.

## 3. Results

### 3.1. Asphyxiating and Irritating Products

Both tested flame retardants demonstrated thermal resistance and did not ignite even at 825 °C. Consequently, it was not possible to replicate the conditions during the fire stages in accordance with ISO 19706 [35]. In Table 2, the average emissions per minute (ppm) of asphyxiant and irritant gases are presented under selected conditions. The primary emission product during the decomposition of the tested fire retardants was carbon monoxide. The amount of released CO increased with rising decomposition temperature. Additionally, during the decomposition of PHT4-DIOL, trace amounts of hydrogen cyanide were detected in the emitted gases, particularly at 650 °C and 825 °C. It may therefore be cautiously suggested, based on the results obtained for the primary asphyxiant and irritant gases analyzed, that fire-retardant agents do not significantly increase the formation of the toxic combustion products included in the scope of the present study. However, this conclusion should be interpreted with caution. Hydrogen bromide (HBr) and bromine radicals, which are well-known major decomposition products of brominated flame retardants, were not included in the toxicity assessment. These species may substantially contribute to the overall toxicity of emitted gases and smoke; therefore, their influence cannot be excluded.

However, when considering the values presented in Table 3, a significant influence of the fire-retardant agents on both the types and quantities of asphyxiant and irritant gases emitted from epoxy resin samples is evident. For these materials, the specimens underwent flaming combustion at 650 °C. The presented results, therefore, reflect emissions that may occur during combustion associated with Fire Stage 2.

To evaluate the influence of individual flame-retardant additives on the toxicity of gases and smoke emitted during combustion, the Fractional Effective Dose (FED) parameter was introduced as a quantitative measure of overall toxic potency. In the mixture of gases and smoke emitted during combustion, epoxy resin samples without brominated flame-retardant additives produced substantial quantities of carbon monoxide, nitrogen oxides (NO and NO_2_), and hydrogen cyanide, resulting in an FED value of 0.425. Although the individual flame retardants alone exhibited FED values below thresholds associated with immediately life-threatening conditions, all epoxy-based materials exceeded the FED = 0.3 criterion, which is commonly regarded as the upper limit for safe evacuation conditions [39].

The introduction of DBDPE into the resin significantly modified the composition of emitted gases. While carbon monoxide emissions increased moderately, concentrations of several irritant and asphyxiant gases, such as NO and SO_2_, decreased. However, NO_2_ levels rose sharply to 61 ppm. As a result of these competing effects, the overall FED increased to 0.974, indicating a substantially elevated risk of incapacitation compared with the unmodified resin. In contrast, samples containing PHT4-DIOL exhibited markedly higher CO, NO_2_, and SO_2_ emissions, resulting in an FED value of 0.898. Although hydrogen cyanide emissions were slightly lower than in the unmodified resin, the increased contribution of oxidative irritants shifted the overall toxicological profile toward more severe respiratory impairment. This FED level exceeds 0.8, a range in which occupants may still survive but face a high probability of unconsciousness, impaired self-evacuation, and increased risk of fatal outcomes.

It is important to emphasize that the calculated FED values do not account for hydrogen bromide, which may be released in significant quantities during combustion and would further increase the overall toxic hazard.

**Figure 2:**
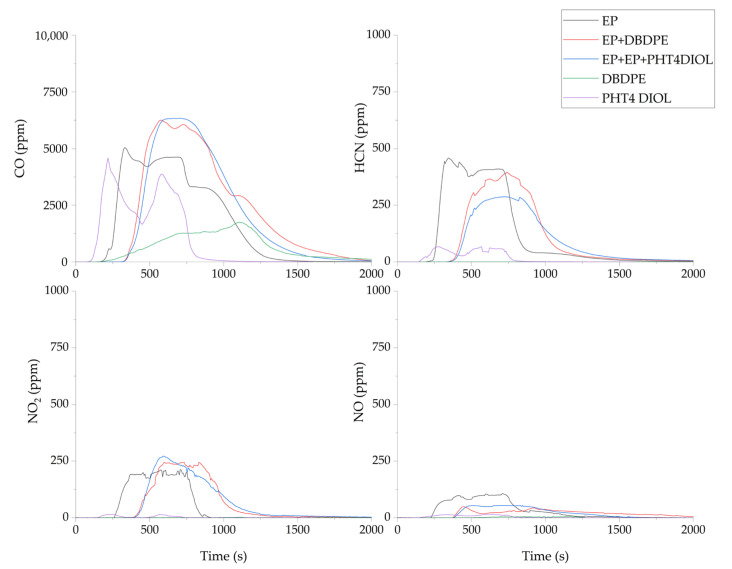
Representative CO, HCN, NO_2_ and NO emission profiles observed during the thermal degradation and combustion of tested materials at 650 °C.

Naturally, the incorporation of flame-retardant additives also influenced the ignition time of the epoxy resin samples, as evidenced by the representative emission profiles of the main asphyxiant and irritant gases presented in Figure 2.

### 3.2. Light Hydrocarbon Products

Another important group of combustion products is light hydrocarbons. Monitoring their emissions is crucial because they provide valuable information about the combustion efficiency and the material’s thermal decomposition pathways. Light hydrocarbons are often formed during incomplete combustion processes, and their presence indicates oxygen-limited or non-ideal burning conditions. Moreover, many of these compounds contribute to the overall flammability of the fire effluent and may act as precursors to secondary toxic species. Continuous monitoring of light hydrocarbons, therefore, enables a more comprehensive assessment of the development of fire hazards and improves understanding of how flame-retardant additives influence decomposition mechanisms [40]. Both brominated additives decomposed with negligible hydrocarbon emissions. In the case of DBDPE, only trace amounts of ethylene were detected under all tested conditions, whereas PHT4-DIOL generated minor quantities of methane, ethane, and ethylene at 650 °C (Table 4).

In contrast, fire effluents from epoxy resin and resin containing flame-retardant additives included methane, ethane, and ethylene. The incorporation of PHT4-DIOL did not significantly affect hydrocarbon emissions. However, the addition of DBDPE resulted in a noticeable reduction in methane, ethane, and ethylene concentrations, attributable to its gas-phase flame-retardant mechanism. The release of bromine radicals inhibits chain reactions, lowers flame temperature, and limits the formation of light hydrocarbons [41]. This behavior can be further rationalized by considering the reactions that occur during thermal decomposition. During the pyrolysis of polymeric matrices, various radicals (e.g., •H, •CH_3_, •CH_2_CH_3_) can be generated via chain scission of polymer segments. These reactive species can effectively scavenge bromine radicals, thereby forming brominated products such as HBr, CH_3_Br, and CH_3_CH_2_Br [18,39]. Such radical trapping reactions reduce the availability of reactive species in the gas phase, thereby suppressing radical chain propagation and contributing to the observed decrease in light hydrocarbon formation. In contrast, PHT4-DIOL acts mainly in the condensed phase and therefore does not significantly affect gas-phase hydrocarbon emissions.

### 3.3. Volatile and Semi-Volatile Organic Products

The application of gas chromatography–mass spectrometry to analyze gases emitted during material decomposition expanded the number of identified chemical substances. Both fire retardants degraded at elevated temperatures, releasing hydrogen bromide as the dominant gaseous products, indicating extensive cleavage of C–Br bonds during thermal degradation.

DBDPE did not degrade at 350 °C. At 650 °C, it degraded, releasing a gas mixture containing volatile halogenated hydrocarbons and higher-molecular-weight polybrominated aromatic halogenated compounds, which tend to condense onto soot (Table 5). The results obtained confirm the literature data [18,19] on the pyrolysis of DBDPE, indicating that DBDPE decomposes between 400 and 600 °C, whereas at lower temperatures it volatilizes. At high temperatures, DPDPE debromination and depolymerization are the dominant processes, leading to the formation of polybrominated biphenyl compounds and highly reactive bromine radicals (Br). It is also possible that the analytical method used was insufficiently sensitive or selective to detect compounds in this group.

PHT4-DIOL is a brominated diol containing reactive functional groups, and thus undergoes a more complex degradation pathway than DBDPE. Consequently, the mixture of gases and smoke emitted during its decomposition contains not only HBr but also oxidation and degradation products originating from the polyol structure. The major decomposition products of PHT4-DIOL were brominated hydrocarbons and brominated phenols (Table 6), which are characterized by high toxicity, environmental persistence, and potential for bioaccumulation [42]. Flameless pyrolysis at 350 °C produced mainly 1,2,4,5-tetrabromobenzene, 1,5-dibromo-2,6-bis(bromomethyl)naphthalene, and 4,5,6,7-tetrabromo-1,3-isobenzofuran-1,3-dione. The first two compounds remained dominant under other degradation conditions. At 650 °C, significant amounts of 1,2,4-tribromobenzene were detected, whereas hydrogen bromide became the predominant gaseous product at 825 °C. PHT4-diol, containing hydroxyl groups and available hydrogen, promotes elimination reactions leading to HBr formation. In contrast, DBDPE primarily undergoes homolytic cleavage of C–Br bonds, generating bromine radicals and debromination products during high-temperature decomposition [14].

The incorporation of brominated flame retardants significantly modified the thermal decomposition pathway of the epoxy resin and altered the composition of the emitted fire effluents, as shown in Table 7.

**Figure 3:**
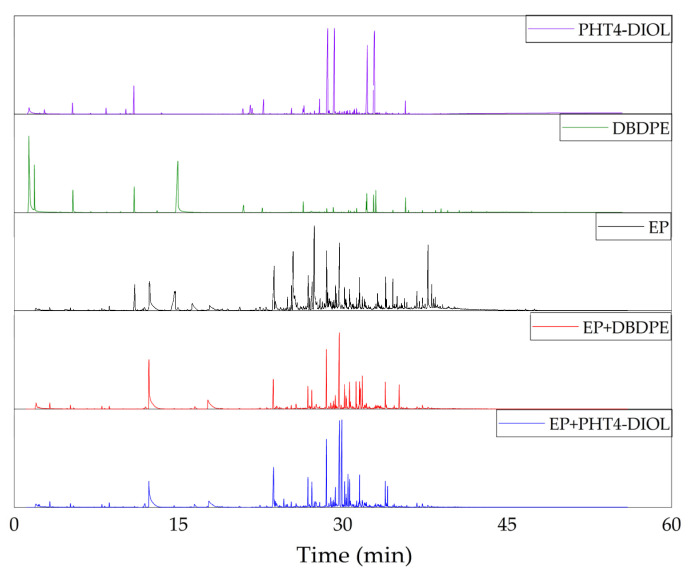
Representative chromatograms of gaseous products emitted during the combustion of flame-retarded resins and flame retardants.

The example chromatograms shown in Figure 3 clearly demonstrate the influence of flame-retardant additives on both the number and the detected chromatographic peaks. The chromatographic profiles and the numerous peaks confirm that the investigated materials underwent thermal decomposition and combustion, accompanied by the emission of numerous, diverse gaseous products. It should also be noted that not all detected compounds were identified and included in Table 5, Table 6 and Table 7; only the selected and positively identified products are presented.

An interesting observation is that some of the products detected in the fire effluents emitted during the combustion of the unmodified epoxy resin were not observed in the analysis of fire effluents originating from the decomposition of the flame-retarded resin samples. This indicates that flame-retardant additives influenced the thermal degradation mechanism of the polymer matrix.

In the gases emitted during the combustion of EP, the following molecules were detected: pyridine, pyrrole, 2-methyl-1H-pyrrole, 3-methylpyridine, and N-(phenylmethylene)methanamine. These compounds were formed by the decomposition of amine-derived fragments in the resin network, as well as by secondary reactions with epoxy and carbonyl fragments [43]. In fire effluents emitted during the combustion of bromine-flame-retarded epoxy resins, nitrogen-containing heterocyclic products (such as pyridine, pyrrole, etc.) were not detected. This is most likely because bromine acts in the gas phase as a radical inhibitor, thereby lowering the temperature and shortening the reaction time required to aromatize amine- and epoxy-derived fragments.

In the gaseous mixture emitted during the combustion of unmodified epoxy resin, many typical decomposition products of the resin were also detected, such as benzene, toluene, phenol, as well as compounds belonging to the group of polycyclic aromatic hydrocarbons (PAHs) [43,44]. These species were formed through thermal decomposition of the bisphenol A-based polymer network, followed by radical-driven fragmentation, dealkylation, and high-temperature aromatization and condensation reactions in the flame zone.

In the case of introducing brominated flame retardants into epoxy resin, aromatic radicals originating from the polymer structure undergo rapid reactions with bromine radicals (Br•) and/or other active bromine species (Br_2_/HBr), leading to the formation of stable Ar–Br bonds. As a result, compounds such as 1,2-dibromobenzene, 1,2-dibromo-2-methylbenzene, 1,2,4-tribromobenzene, 2-bromonaphthalene, 5-bromo-1,2-dihydroacenaphthalene, 2,4,4′-tribromodiphenyl ether, and 1,2,4,5-tetrabromobenzene were detected.

Bromobenzene and dibromobenzene were detected exclusively in the EP + PHT4-DIOL system because this flame retardant readily releases reactive bromine species (Br•, HBr) into the gas phase, which efficiently brominate small aromatic decomposition products of the epoxy resin. In contrast, DBDPE, which is more thermally stable, releases bromine much more slowly.

On the other hand, highly brominated aromatic compounds such as brominated alkylbenzenes, tribromobenzenes, tetrabrominated benzenes, and bromonaphthalene were formed in both DBDPE- and PHT4-DIOL-containing epoxy systems because they originate from high-temperature flame-zone reactions between commonly generated aromatic pyrolysis products and reactive bromine species, making their formation largely independent of the specific flame retardant precursor.

It should be emphasized that among these compounds, brominated diphenyl ethers are of particular concern due to their persistence, potential for bioaccumulation, and endocrine-disrupting properties. In contrast, lower-brominated aromatics, such as dibromobenzenes, contribute more significantly to acute toxicity [42,44].

## 4. Discussion

The present results demonstrate that the fire performance of brominated flame retardants cannot be evaluated solely by ignition resistance or flame-retardant efficiency. Although both DBDPE and PHT4-DIOL exhibited high thermal stability and did not sustain flaming combustion up to 825 °C, their incorporation into epoxy resin significantly influenced the composition and toxicity of the resulting fire effluents, indicating a trade-off between reduced flammability and increased combustion hazard.

FED analysis showed a clear increase in overall toxic potency for flame-retarded systems. Non-modified epoxy resin produced an FED value of 0.425, whereas the incorporation of DBDPE and PHT4-DIOL increased FED values to 0.974 and 0.898, respectively. These results indicate conditions associated with severe impairment of evacuation capability. It should be noted that hydrogen bromide was not included in the FED calculation, suggesting that the total toxic hazard may be underestimated.

The results demonstrate that incorporating brominated flame retardants significantly alters the thermal degradation pathways of epoxy resin and, consequently, changes the composition of the emitted fire effluents. The decomposition of unmodified epoxy resin produced a broad range of hydrocarbons, nitrogen-containing heterocyclic compounds, phenols, and polycyclic aromatic hydrocarbons (PAHs), arising from fragmentation and secondary aromatization within the bisphenol A-based polymer network. In contrast, the presence of brominated flame retardants promoted the formation of brominated aromatic compounds and reduced the emission of light hydrocarbons such as methane, ethane, and ethylene. This effect was particularly pronounced for DBDPE and can be attributed to its gas-phase flame-retardant mechanism, in which bromine radicals act as radical scavengers, suppressing chain propagation reactions and limiting hydrocarbon formation. Simultaneously, interactions between bromine species and aromatic radicals generated during epoxy decomposition resulted in the formation of brominated benzenes, bromophenols, brominated naphthalene derivatives, and brominated diphenyl ethers. Similar observations have been reported in previous studies [43,44,45,46] on brominated epoxy systems, in which hydrogen bromide and brominated aromatic compounds were identified as key thermal degradation products generated via radical bromination and destabilization of the epoxy network.

The results also indicate substantial differences in the degradation behavior of the investigated flame retardants. DBDPE exhibited relatively high thermal stability and decomposed primarily at elevated temperatures via debromination and depolymerization, generating brominated aromatic products and bromine radicals. This behavior is consistent with recent mechanistic studies demonstrating that DBDPE decomposition proceeds via cleavage of C–Br bonds and the formation of bromine radicals, hydrogen bromide, and lower-molecular-weight brominated aromatic species [18,41]. In contrast, PHT4-DIOL underwent a more complex degradation pathway due to the presence of reactive hydroxyl groups, producing brominated hydrocarbons and aromatic compounds.

Furthermore, some nitrogen-containing compounds detected during decomposition of the unmodified epoxy resin were absent in the fire effluents from brominated systems, suggesting that bromine species altered secondary gas-phase reactions and inhibited the formation of heterocyclic nitrogen compounds. Similar suppression of nitrogen-containing pyrolysis products and extensive formation of organobrominated compounds under oxidative and pyrolytic conditions have also been described for brominated epoxy materials and electronic waste polymers [18,46].

The methodological approach combining real-time FT-IR analysis of combustion gases with SPME-GC-MS identification of VOCs and SVOCs, together with FED-based toxicity assessment, provided a comprehensive evaluation of fire effluent composition. The use of a steady-state tube furnace enabled the simulation of fire conditions (developed fire stage), offering greater relevance than conventional pyrolysis-based tests. Although the tube furnace allows for comparable and reproducible studies of decomposition product emissions, it represents a simplified model of real fire conditions; therefore, the results should be interpreted with these limitations in mind. An interesting extension of the study would be to conduct future investigations on a larger scale, for example, using furnace tests following the ISO 834 standard fire curve [47]. Such an approach would allow the tested materials to be exposed to fire from three sides under more realistic thermal conditions. Additionally, this method enables continuous monitoring of the temperature inside the material using thermocouples positioned at different depths, allowing for the evaluation of the temperature gradient within the tested element. Such experiments could provide valuable complementary information on thermal degradation processes and fire behavior under conditions closer to real fire scenarios, especially when supplemented with scanning electron microscopy (SEM) and X-ray diffraction (XRD) analyses to characterize structural and morphological changes in the materials after thermal exposure [48].

Overall, the results indicate that brominated flame retardants significantly modify thermal degradation pathways of epoxy resins, leading to more complex combustion products and increased toxicological burden. These findings highlight the need to consider both flammability and fire effluent toxicity as complementary parameters when assessing flame-retarded materials.

## 5. Conclusions

Brominated flame retardants (DBDPE and PHT4-DIOL) did not sustain combustion under the conditions investigated; however, their incorporation into epoxy resin significantly altered the composition and toxicity of fire effluents. A clear trade-off was observed between improved flammability resistance and increased combustion toxicity.

Fire toxicity assessment using the FED model indicated a substantial increase in overall hazard for flame-retarded systems compared to unmodified epoxy resin, suggesting that conventional flammability-based evaluation may not adequately reflect the total fire risk.

The applied integrated methodology, combining FT-IR, SPME-GC-MS, and FED analysis under realistic fire conditions, enabled a more comprehensive characterization of combustion behavior and emission profiles.

Overall, the results highlight that flame retardancy and fire effluent toxicity should be considered as complementary but independent parameters in the assessment of polymeric materials.

## Figures and Tables

**Table 1:** Steady-state tube furnace test data for all materials.

Material	Fire Stage	Temp of Furnace [°C]	Primary/Secondary Air Flow Rate [L/min]	Total Mass Sample [g]	SPME Sampling Period [s]	Observations of Burning Behavior
DBDPE	1b	350	2/48	20.2 ± 0.2	650–950	Non-flaming
	2 *	650	10/40	20.7 ± 0.2	650–950	Non-flaming
	2 *	825	10/40	20.7 ± 0.2	650–950	Non-flaming
PHT4-DIOL	1b	350	2/48	20.0 ± 0.2	650–950	Non-flaming
	2 *	650	10/40	20.0 ± 0.2	600–900	Non-flaming
	2 *	825	10/40	20.3 ± 0.2	600–900	Non-flaming
EP	2	650	10/40	39.4 ± 0.5	550–850	Flaming D_O2_ = 3.07%
EP + DPDPE	2	650	10/40	46.6 ± 0.5	550–850	Flaming D_O2_ = 2.77%
EP + PH4-DIOL	2	650	10/40	35.8 ± 0.5	550–850	Flaming D_O2_ = 2.77%

* The tested materials did not undergo flaming combustion, and therefore, it was not possible to replicate Fire Stage 2 of fire development in accordance with the standard.

**Table 2:** Average emissions per minute (ppm) for carbon monoxide (CO), nitrous oxide (N_2_O), nitrogen monoxide (NO), nitrogen dioxide (NO_2_), formaldehyde (CH_2_O), and hydrogen cyanide (HCN) during thermal degradation of brominated flame retardants.

Flame Retardant	Fire Stage	Temp. Decomp. (°C)	Average Emissions per Minute (ppm)						
			CO	N_2_O	NO	NO_2_	SO_2_	CH_2_O	HCN
DBDPE	1b	350	0.57 ± 0.1	-	-	-	7.09 ± 1.84	-	-
	2 *	650	319 ± 19	-	0.68 ± 0.11	0.29 ± 0.05	50.2 ± 10.5	-	-
	2 *	825	764 ± 61	-	2.19 ± 0.41	0.17 ± 0.03	66.1 ± 8.6	-	-
PHT4-DIOL	1b	350	4.87 ± 0.6	-	-	2.26 ± 0.54	25.1 ± 4.5	-	-
	2 *	650	460 ± 34	-	1.74 ± 0.31	1.01 ± 0.23	1.55 ± 0.54	-	7.23 ± 2.1
	2 *	825	630 ± 83	-	4.13 ± 0.87	-	-	-	7.23 ± 2.7

* The tested materials did not undergo flaming combustion, and therefore, it was not possible to replicate Fire Stage 2 of fire development in accordance with the standard.

**Table 3:** Emission yields (g/kg) of carbon monoxide (CO), nitrous oxide (N_2_O), nitrogen monoxide (NO), nitrogen dioxide (NO_2_), formaldehyde (CH_2_O), and hydrogen cyanide (HCN) during combustion of brominated epoxy resins.

Material	Average Emissions per Minute (ppm)								FED
	CO	N_2_O	NO	NO_2_	SO_2_	NH_3_	CH_2_O	HCN	
EP	54 ± 49	7.68 ± 7.6	39.3 ± 6.2	5.31 ± 0.8	20.9 ± 12	-	4.08 ± 1	63.1 ± 18	0.425
EP + DPDPE	673 ± 87	0.85 ± 0.1	12.4 ± 1.6	61 ± 12	4.80 ± 1.1	-	3.04 ± 0.5	59.9 ± 14	0.974
EP + PHT4-DIOL	810 ± 65	0.95 ± 0.1	16.7 ± 3.2	82 ± 18	8.43 ± 1.9	-	3.85 ± 1	66.6 ± 21	0.898

**Table 4:** Average emissions per minute (ppm) for light hydrocarbons during thermal degradation of brominated flame retardants and brominated epoxy resins.

Flame Retardant	Fire Stage	Temp. Decomp. (°C)	Average Emissions per Minute (ppm)				
			CH_4_	C_2_H_6_	C_2_H_4_	C_3_H_8_	C_6_H_14_
DBDPE	1b	350	-	-	1.15 ± 0.2	-	-
	2 *	650	-	-	3.08 ± 0.5	-	-
	2 *	825	-	-	2.57 ± 0.6	-	-
PHT4-DIOL	1b	350	-	2.0 ± 0.1	0.24 ± 0.1	1.77 ± 0.4	-
	2 *	650	27 ± 1.2	1.2 ± 0.1	9.08 ± 1.5	0.51 ± 0.2	-
	2 *	825	1.1 ± 0.1	0.34 ± 0.05	0.14 ± 0.05	-	-
EP	2	650	77 ± 6	30 ± 0.3	12 ± 2.1	-	-
EP + DPDPE	2	650	36 ± 2	12 ± 0.9	3.8 ± 0.1	-	-
EP + PHT4-DIOL	2	650	71 ± 4	25 ± 2.4	12 ± 4.3	-	-

* The tested materials did not undergo flaming combustion, and therefore, it was not possible to replicate Fire Stage 2 of fire development in accordance with the standard.

**Table 5:** List of chemical substances identified in the mixture of gases and smokes formed during the thermal decomposition of DBDPE using the GC-MS method.

Nr.	Retention Time (min)	Identified Chemical Products	CAS	Amount (%)		
				350 °C	650 °C	825 °C
1	1.48	Hydrogen bromide	10035-10-6		32.79	15.33
2	1.84	Bromine	7726-95-6		6.44	25.41
3	6.99	Tribromomethane	75-25-2		0.19	
4	9.71	Tribromoethene	598-16-3		0.18	
5	13.05	Tetrabromomethane	558-13-4		0.48	
6	22.65	Tetrabromoethene	79-28-7		1.03	
7	28.54	1,2,4-Tribromobenzene	615-54-3		0.48	
8	30.52	1,2,4,5-Tetra(bromomethyl)-benzene	15442-91-8		0.28	
9	32.14	1,2,4,5-Tetrabromobenzene	636-28-2		1.38	
10	35.73	1,5-Dibromo-2,6-bis(bromomethyl)naphthalene	85477-63-0		1.83	

**Table 6:** List of chemical substances identified in the mixture of gases and smokes formed during the thermal decomposition of PHT4-DIOL using the GC-MS method.

Nr.	Retention Time (min)	Identified Chemical Products	CAS	Amount (%)		
				350 °C	650 °C	825 °C
1	1.36	Propanal	123-38-6	3.40		
2	1.45	2-Propen-1-ol	107-18-6	1.37		
3	1.48	Hydrogen bromide	10035-10-6	3.40	0.15	58.17
4	2.26	Bromomethane	74-83-9		0.35	
5	2.76	Dibromomethane	74-95-3		0.62	
6	3.45	2-Bromoethanol	540-51-2	0.56		
7	3.83	Bromoacetone	598-31-2		0.59	
8	4.02	1-Bromo-2-propanol	19686-73-8	2.12		
9	4.48	3-Pentanol	584-02-1	1.00		
10	6.97	Tribromomethane	75-25-2		0.11	0.70
11	8.39	2,3-Dibromo-1-propene	513-31-5		0.22	
12	8.67	Bromobenzene	108-86-1		0.77	
13	9.96	Tribromoethene	598-16-3		0.09	
14	10.19	Phenol	271-89-6		0.65	
15	10.39	Propylene Carbonate	108-32-7	0.58		
16	13.45	2-Bromophenol	4265-25-2		0.25	
17	19.09	1,1′-Oxybis [2-bromoethane]	5414-19-7	1.88		
18	21.40	Naphthalene	91-20-3		0.23	
19	21.56	1,4-Dibromobenzene	106-34-6		1.99	
20	22.56	Tetrabromoethene	79-28-7		0.44	
21	24.67	5-Bromobenzofuran	23145-07-5		0.09	
22	25.31	3-Bromophenol	1836-06-2		0.69	
23	26.46	2,4-Dibromophenol	54965-04-7		1.19	
24	26.78	4,5-Dibromo-3(2H)-pyridazinone	5788-58-9		0.14	
25	27.02	1-Bromo-2-(2-methoxyethoxy)ethane	54149-17-6	1.88		
26	27.28	1,1′-Oxybis [2-(2-bromoethoxy)ethane]	31255-26-2	0.51		
27	28.17	2,5-Dibromo-3,6-dimethylbenzonitrile	38319-75-4		0.13	2.29
28	28.52	1,2,4-Tribromobenzene	615-54-3	0.66	19.82	
29	28.70	2-Bromonaphthalene	580-13-2		0.26	
30	29.31	Dibenzofuran	132-64-9		0.34	
31	30.75	1,2,4-Tribromo-5-methylbenzene	3278-88-4			0.55
32	30.89	2,4,6-tribromophenol	118-79-6		0.15	
33	32.24	1,2,4,5-Tetrabromobenzene	636-28-2	9.69	11.12	0.57
34	32.32	1,4-Dibromonaphthalene	83-53-4		0.14	
35	33.19	2,4,6-Tribromobenzene-1,3-diol	2437-49-2		0.15	
36	34.21	2,4,4′-Tribromodiphenyl ether	41318-75-6	0.48		
37	34.98	3,4,5,6-Tetrabromo-o-xylene	NIST 291142	0.65		
38	35.09	2,3,5,6-Tetrabromo-p-xylene	23488-38-2	0.26	0.09	
39	35.53	3,6-Dibromobenzo[e]pyrene	77508-03-3		0.91	
40	35.71	1,5-Dibromo-2,6-bis(bromomethyl)naphthalene	85477-63-0	6.08	1.15	0.73
41	36.70	2,3,4,5,6-Pentabromobenzyl alcohol	79415-41-1	0.24		
42	37.11	1,2,3,4-Tetrabromo-5-methoxy-6-methylbenzene	108608-61-3	0.79		
43	37.75	3,4,5-Tribromobenzoic acid	618-74-6	0.86		
44	38.84	4,5,6,7-Tetrabromo-1,3-isobenzofurandione	632-79-1	3.06		

**Table 7:** List of chemical substances identified in the mixture of gases and smokes formed during the thermal decomposition of epoxy resins using the GC-MS method.

Nr.	Retention Time (min)	Identified Chemical Products	CAS	Amount (%)		
				EP	EP + DBDPE	EP + PHT4DIOL
1	2.75	Benzene	71-43-2	0.13	0.68	0.58
2	4.12	Pirydine	110-86-1	0.13		
3	4.51	Pyrrole	109-97-7	0.04		
4	4.64	Toluene	108-88-3	0.11	0.46	0.36
5	4.91	2,4-Pentadienenitrile	1615-70-9	0.06	0.19	0.20
6	6.79	2-Methyl-1H-pyrrole	636-41-9	0.04		
7	7.66	3-Methyl-pyridine	108-99-6	0.05		
8	8.19	Styrene	100-42-5	0.21	0.36	0.47
9	9.44	Bromobenzene	108-86-1			0.09
10	10.52	Benzaldehyde	100-52-7	1.97		0.14
11	11.38	Benzonitrile	100-47-0	0.37	0.57	0.87
12	11.84	Phenol	108-95-2	5.75	17.42	10.54
13	14.14	Benzyl alcohol	100-51-6	3.50		
14	14.45	N-(Phenylmethylene)methanamine	622-29-7	0.49		
15	15.34	Phenyl acetate	122-79-2	0.11		
16	17.35	p-Cresol	106-44-5	1.10	7.23	4.29
17	23.23	Naphthalene	91-20-3	4.26	4.83	6.21
18	24.11	1,4-Dibromobenzene	106-34-6			0.81
19	24.33	4,7-Dimethylbenzofuran	28715-26-6	0.11	0.24	0.29
20	24.83	2-Ethenylbenzofuran	7522-79-4	1.12	0.40	0.49
21	24.97	2,3-Dihydrobenzofuran	496-16-2	5.23		
22	26.31	1,4-Dibromo-2-methylbenzene	615-59-8		2.24	0.33
23	26.35	2-Methylnaphthalene	91-57-6	1.91	1.82	2.52
24	26.91	p-Isopropenylphenol	4286-23-1	7.66		
25	28.83	Biphenyl	92-52-4	1.15	6.15	6.25
26	29.20	Acenaphthylene	208-96-8	4.51	10.32	11.47
27	29.35	1,2,4-Tribromobenzene	615-54-3		0.34	9.89
28	29.49	2-Bromonaphthalene	580-13-2		0.13	0.21
29	29.85	1-Isocyanonaphthalene	9-04-1984	0.32	0.85	0.58
30	30.12	Dibenzofuran	132-64-9	1.02	2.44	0.27
31	30.71	1,2,4-Tribromo-5-methylbenzene	3278-88-4		2.19	0.45
32	31.03	Fluorene	86-73-7	1.22	2.58	2.59
33	32.59	5-Bromo-1,2-dihydroacenaphthylene	2051-98-1		0.46	0.31
34	32.65	p-Hydroxybiphenyl	92-69-3	0.65	0.10	0.44
35	32.95	1,2,4,5-Tetrabromobenzene	636-28-2		0.30	0.18
36	33.40	Phenanthrene	85-01-8	1.23	2.40	0.35
37	34.65	2,4,4′-Tribromodiphenyl ether	41318-75-6		2.49	0.10
38	36.26	Fluoranthene	206-44-0	0.82	0.47	0.44
39	36.77	4,4′-Ethylidenediphenol	5-08-2081	0.72	0.42	

## Data Availability

The original contributions presented in this study are included in the article. Further inquiries can be directed to the corresponding author.
